# Parafoveal Retinal Vascular Response to Pattern Visual Stimulation Assessed with OCT Angiography

**DOI:** 10.1371/journal.pone.0081343

**Published:** 2013-12-02

**Authors:** Eric Wei, Yali Jia, Ou Tan, Benjamin Potsaid, Jonathan J. Liu, WooJhon Choi, James G. Fujimoto, David Huang

**Affiliations:** 1 Casey Eye Institute, Oregon Health & Science University, Portland, Oregon, United States of America; 2 Department of Electrical Engineering and Computer Science, and Research Laboratory of Electronics, Massachusetts Institute of Technology, Cambridge, Massachusetts, United States of America; 3 Advanced Imaging Group, Thorlabs, Inc., Newton, New Jersey, United States of America; University College London, United Kingdom

## Abstract

We used optical coherence tomography (OCT) angiography with a high-speed swept-source OCT system to investigate retinal blood flow changes induced by visual stimulation with a reversing checkerboard pattern. The split-spectrum amplitude-decorrelation angiography (SSADA) algorithm was used to quantify blood flow as measured with parafoveal flow index (PFI), which is proportional to the density of blood vessels and the velocity of blood flow in the parafoveal region of the macula. PFI measurements were taken in 15 second intervals during a 4 minute period consisting of 1 minute of baseline, 2 minutes with an 8 Hz reversing checkerboard pattern stimulation, and 1 minute without stimulation. PFI measurements increased 6.1±4.7% (p = .001) during the first minute of stimulation, with the most significant increase in PFI occurring 30 seconds into stimulation (p<0.001). These results suggest that pattern stimulation induces a change to retinal blood flow that can be reliably measured with OCT angiography.

## Introduction

 The notion that the brain has an intrinsic method to regulate local blood flow in response to a stimulus was first proposed by Roy and Sherrington in 1890 [1]. This phenomenon, known as neurovascular coupling, has since been confirmed by brain researchers who have measured changes in cerebral blood flow in response to a variety of stimuli and mapped out the activity of the brain [2].

As an extension of the central nervous system (CNS), the retina provides a unique opportunity to study the neurovascular coupling phenomenon *in vivo*. Neurovascular coupling in the eye was first confirmed in the cat through increased optic nerve head blood flow in response to flicker light, and was later confirmed in primates and human eyes as well [3]–[5].

Many techniques have been used to evaluate neurovascular coupling in the human retina. Early studies using techniques such as the blue field simulation, pulsed Doppler sonography, and laser Doppler velocimetry (LDV) measured the vascular response to flickering light as a change in blood velocity [5]–[7]. Techniques used later on, including laser Doppler flowmetry, scanning laser Doppler flowmetry (SLDF), and Doppler OCT, were able to measure vascular changes by measuring the flow rate of moving scatters, such as red blood cells (RBC) [6], [8]–[10].

To measure local microcirculation (arterioles, venules, and capillaries) in the eye, we developed OCT angiography using a high speed swept-source OCT device [11]. Rather than evaluating blood flow using phase differences, OCT angiography with split-spectrum amplitude-decorrelation angiography (SSADA) relies on the variation of signal amplitude to extract flow [12].

Unlike Doppler OCT or LDV which measure large retinal vessels (arteries and veins) to obtain total retinal blood flow, OCT angiography with SSADA [12] is capable of measuring both macro- and micro-circulation (down to capillaries). This allows OCT angiography to measure the microcirculation of specific regions of the eye that would not be possible using techniques that measure total retinal blood flow. SLDF with the Heidelberg retina flowmeter (HRF) is also able to measure flow in capillary beds, but the sampling depth is unclear. Therefore, the received signal may not necessarily be isolated from the retina and the resultant flow may be affected by flow from choroidal capillaries [13].

More recent approaches to imaging retinal microcirculation include adaptive optics scanning laser ophthalmoscopy[14]–[18] and adaptive optics optical coherence tomography[19], which also utilize signal variation to infer blood flow and provide direct measurement values for RBC velocity (or variation). Compared to OCT angiography, however, their drawback is an inherently small imaging field of view (less than 800×800 µm^2^). Other techniques using signal variation for blood flow extraction have also been demonstrated recently for functional imaging retinal capillaries, which may also be appropriate for studying the vascular response to visual stimulation [20]–[22].

In this article, we report the first use of OCT angiography with SSADA to measure the change in blood flow to the parafoveal retina when stimulated with an 8 Hz reversing checkerboard pattern.

## Methods

### Study population

The study was performed at the Casey Eye Institute, Oregon Health & Science University (OHSU). The research protocol was approved by the OHSU institutional review board (Approval number: IRB 00008456) and carried out in accordance with the Declaration of Helsinki. Written informed consent was obtained from each subject following an explanation of the nature of the study. Five healthy volunteers, 4 male and 1 female (mean age 35±9.9 years) participated in the study. Measurements were obtained on one eye for all five subjects.

### Experimental design

Each subject was dilated with 1% tropicamide and 2.5% phenylephrine eye drops 20–40 minutes prior to OCT scanning. They were scanned in the seated position in front of the OCT scanner with their heads stabilized with a supporting chin rest and forehead rest. Subjects were instructed to fixate upon an internal fixation target - a small red dot (0.05°), which was projected by an attenuated pico projector using digital light processing technology (Texas Instruments, Dallas, TX, USA). The experimental sequence consisted of 1 minute of baseline measurements proceeded by 2 minutes of stimulation measurements and 1 minute of post-stimulation measurements following cessation of the pattern. A total of 16 OCT scans taken in 15 second intervals were obtained during each 4 minute session. The experimental sequence was performed twice for each subject, with a 10 minute rest period between sequences.

### Pattern stimulus

**Figure 1:**
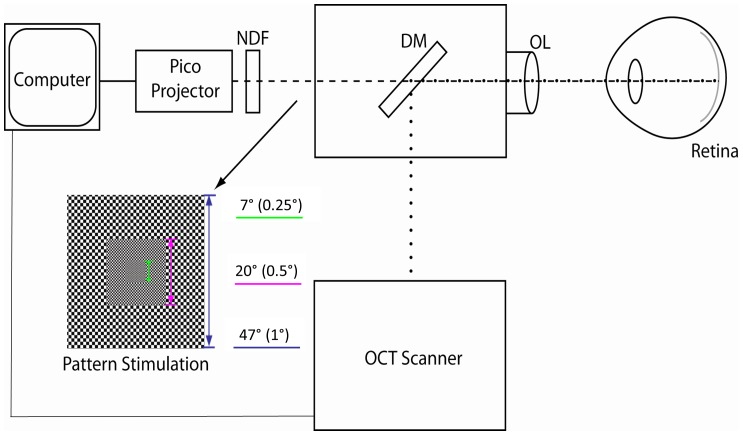
Experiment setup of the pattern stimulation apparatus mounted behind the OCT scan head. Size of the region (size of the checkerboard square size) was shown beside the pattern stimulation. NDF, Neutral density filter; DM, Dichroic mirror; OL, Objective lens.

The pattern stimulus was generated in Matlab R2012b (MathWorks, Natick, MA, USA) and sent to the same pico projector mounted behind the OCT scan head used for delivering the fixation target (Fig. 1). A neutral density filter was placed in the optical path to reduce the luminance of the stimulus pattern on the corneal plane to 0.035 lumen/cm^2^. According to the ANSI safety limit [23], at visible wavelengths, the maximum permissible 2 minute exposure is 0.05 W/cm^2^ = 33.4 lumen/cm^2^ (using 1 Watt = 668 lumen) on the cornea. In this study, the maximum ocular exposure was well below the ANSI limit.

Our stimulation pattern was similar to the alternating checkerboard used in electroretinography which measures the electrical response of retinal ganglion cells [24]. We modified the pattern to have smaller checkerboard squares towards the center (Fig. 1) so that the spatial frequency of stimulation was higher in the central macula where the cone density is higher. The width of three regions in the checkerboard was 47°, 20° and 7° visual angles; and the checkerboard square sizes within the region were 1°, 0.5° and 0.25°, respectively. The pattern reversal frequency was 8 cycles/second (square-wave) which had been shown to give a robust vascular response in larger retinal vessels [25].

### System and scan

A custom built OCT device was used for this study. The device operated at an axial scan speed of 100 kHz using a swept source cavity laser operating at 1050 nm with a tuning range of 100 nm. With this configuration, a resolution of 5.3 µm axially and 18 µm laterally at an imaging depth of 2.9 mm in tissue was achieved.

A 3×3 mm scanning area with 8° visual angle centered on the fovea was captured for blood flow measurements. In the fast transverse (X) direction, 200 axial scans were sampled along a 3 mm region to obtain a single B-scan. Eight consecutive B-scans (M-B scans) were captured at a fixed Y position before proceeding to the next sampling location. A total of 200 locations along a 3 mm region in the slow transverse (Y) direction were sampled to form a 3-D data cube. With a B-scan frame rate of 476 frames per second, the 1,600 B-scans in each scan were acquired in approximately 3.4 seconds.

### Quantification of blood flow with parafoveal flow index

The split-spectrum amplitude-decorrelation angiography (SSADA) algorithm was used to distinguish vessels from static tissue [12]. As seen in real-time OCT reflectance images, the amplitude of signal returning from nonstatic tissue varies rapidly over time [26]. By calculating the decorrelation (D) of signal amplitude from consecutive B-scans, a contrast between static and nonstatic tissue was created that allowed for the visualization of blood flow in the form of an angiogram. However, decorrelation can also be generated through bulk motion. To reduce this effect, the SSADA algorithm split the spectrum and thereby lengthened the axial resolution element, which minimized axial motion noise due to orbital pulsation (bulk motion noise along the axial direction). Furthermore, the algorithm incorporated two steps to further remove background tissue motion and saccadic motion artifacts. First, the median decorrelation (an estimate of bulk motion effect) was calculated for each average decorrelation frame and then subtracted from it. This sets the decorrelation value for bulk tissue to around zero. Second, using outlier analysis, decorrelation frames with excessive median decorrelation values (i.e., frames corrupted by saccadic and micro-saccadic eye movements) were removed and replaced by the average of neighboring frames.

Physical flow phantom calibration experiments have been performed in our research group [27] and by others [28]. Decorrelation can be considered as a metric for measuring fluctuation in backscattered OCT signal amplitude (intensity) that does not depend on the average signal level. More specifically, blood flow results in fluctuations in the amplitude of OCT fringes (speckle) as RBCs move within a particular voxel. Therefore, the eight M-B frames contain fluctuating values of OCT output intensities at any given voxel in the flow of blood, and the definition of decorrelation is constructed so that fluctuating intensities yield high decorrelation values (approaching 1.0). Pixels in the M-B frames that contain static tissue and hence constant intensities yield small decorrelation values (approaching 0). The faster blood particles move across the laser beam, the higher decorrelation of the received signals within a velocity range set by the scan parameters. In the other words, decorrelation is approximately linear to flow velocity (the distance traveled by RBCs flowing across light beam within a unit time) [12], [27], [28]. However, beyond a saturation velocity that is defined by the time interval between consecutive OCT M-B frames, decorrelation increases more slowly with velocity and eventually reaches an upper bound [12], [28]. This saturation velocity should be approximately 0.3 to 0.7 mm/sec according to our and others' physical phantom experiments, accounting for our wavelength of 1050 nm and inter-MB frame interval of 2 msec [28].

**Figure 2:**
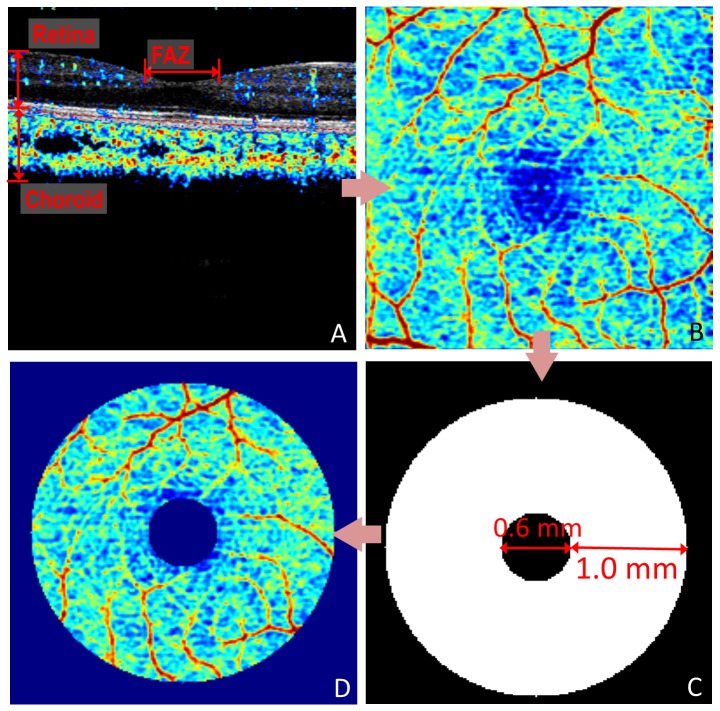
Procedural flow chart for quantification of parafoveal blood flow. (A) Separation of retinal flow from choroidal flow using the RPE plane (dashed line) as the dividing boundary. (B) Retinal flow projected in an *en face* projection of maximum decorrelation. (C) Mask defining the region of the parafoveal retina. (D) Isolated parafoveal retinal flow following overlay of mask on the *en face* angiogram.

In order to eliminate the influence of blood flow from the underlying choroid, segmentation was performed to isolate retinal blood flow using the retinal pigment epithelium (RPE) as the dividing boundary between the retina and the choroid [29] (Fig. 2). Isolation of blood flow in the retinal layers was performed by transferring the RPE detected in structural images to corresponding angiography images. The *en face* X-Y maximum projections of angiography were formed by selecting the value of greatest decorrelation within each axial coordinate of the segmented retina volume.

A masking procedure was performed to obtain flow information from the site of the parafoveal retina. The masking overlay consisted of an annulus with a width of 1 mm defined by an inner radius of 0.3 mm and an outer radius of 1.3 mm. Regions within the area of the annulus were assigned a value of 1 and regions outside the area of the annulus were assigned a value of 0. The mask was overlaid onto the *en face* angiograms with the annulus centered on the center of the foveal avascular zone (FAZ). The FAZ was defined as the region of the macula in the *en face* image void of blood vessels, including capillaries. By multiplying the mask with the original angiography projection, a localized flow map of the parafoveal retina was created. Parafoveal flow index (PFI) measurements were calculated as the average decorrelation value in the localized parafoveal region (A) given by,
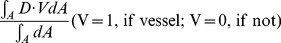
(1)where *D* is the decorrelation value acquired by SSADA. The threshold D value used to judge *V* as 1 or 0 was set at 0.125, two standard deviations above the mean decorrelation value in the noise region – FAZ. Flow index is a dimensionless parameter between 0 and 1 that is proportional to the density of blood vessels (fractional area occupied by vessels) and the velocity of blood flow in the parafoveal region.

In order to evaluate the remaining noise above the threshold (D = 0.125) on the flow map, foveal flow index (FFI) measurements were also performed in the FAZ with a radius of 0.3 mm. Since there is no retinal circulation in the FAZ, the FFI is an assessment of background motion noise.

### Statistical analysis

Two-tailed paired t-tests were used to compare PFI in stimulation and post-stimulation periods (12 time points) with averaged baseline PFI. Because the outcome measures were tested against 12 hypothesized predictors, a Bonferroni-adjusted significance level of 0.0042 was calculated to account for the increased possibility of Type-I error.

Two tailed paired t-tests were used to compare FFI (noise) at 30 sec into stimulation and 30 sec after stimulation with FFI at 30 sec baseline. Bonferroni-adjusted significance level of 0.025 was used for the significance level. Repeatability was expressed as pool coefficient of variation (CV) (the ratio of pool standard deviation from all subjects and mean). It was calculated using consecutive baseline measurements made during the same experimental sequence as well as baseline measurements made between two experimental sequences.

## Results

### Repeatability

The repeatability of consecutive baseline measurements taken within a single experimental sequence was 1.3% CV. The repeatability between two baseline sequences was 2.1% CV.

### Assessment of Background Motion Noise using Foveal Flow Index

**Figure 3:**
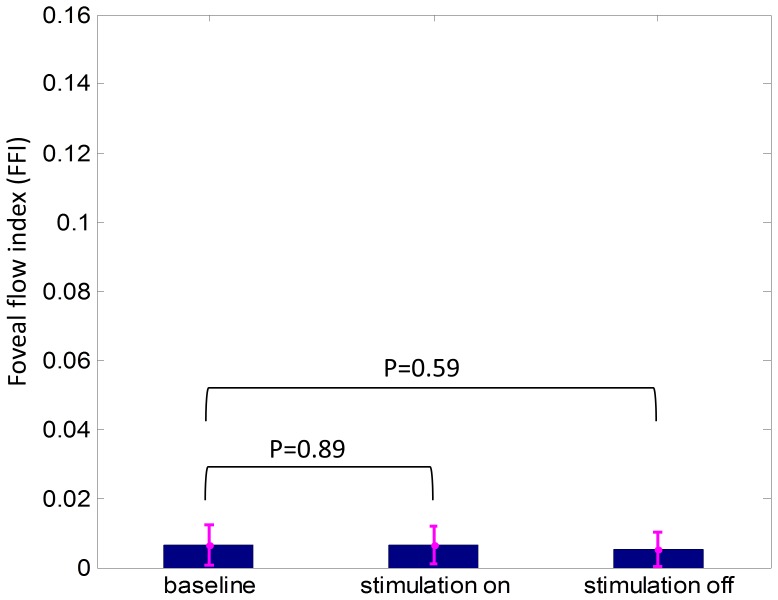
Foveal flow index (FFI) representing the noise analyzed at 30 sec of baseline, stimulation and post-stimulation period. The FFI of both stimulation and post-stimulation was not significantly different from baseline. Two tailed paired t-tests with Bonferroni correction were used.

The flow indexes within the FAZ were calculated for all experiment sequences of five subjects at 30 sec of baseline, stimulation and post-stimulation. Figure 3 shows that the noise at selected time points was consistently low (∼0.006). Compared to baseline, the increase of FFI was not significant at 30 sec into simulation (p = 0.89) and the decrease of it was not significant at 30 sec after stimulation (p = 0.59).

### Parafoveal angiograms

**Figure 4:**
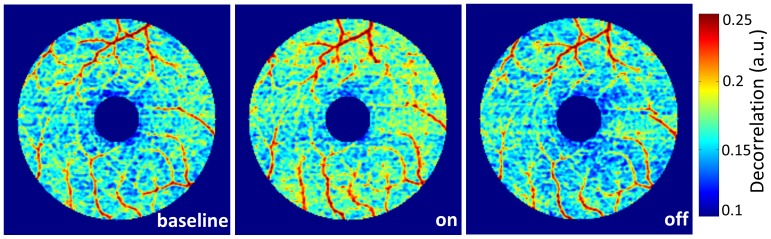
False color representation of *en face* retinal angiograms captured during the course of the experiment. Increased flow (warmer color - higher decorrelation values) was seen in the angiogram captured 30 seconds after the visual stimulation was turned on (middle) compared to baseline (left). The angiogram captured 30 seconds after stimulation was turned off (right) did not appear different from baseline.

The *en face* angiograms (Fig. 4) show increased perfusion after visual stimulation, and a return to baseline level after the stimulation was turned off.

### Parafoveal flow index

**Figure 5:**
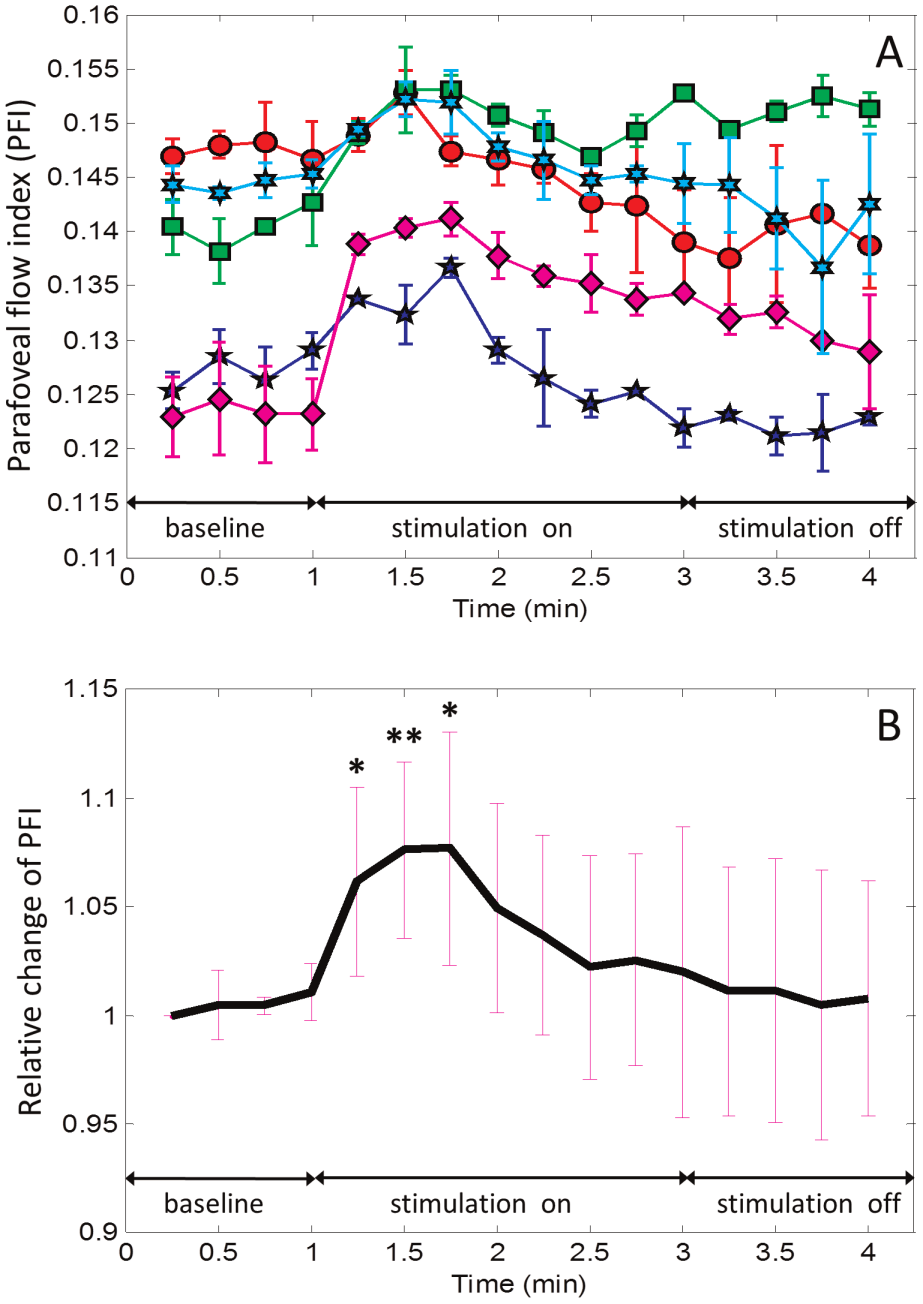
Time course of parafoveal flow index (PFI) with stimulation turned on and then off. (A) Plot for individual subjects averaged from two experimental sequences. (B) Relative change from baseline averaged over all subjects. Two tailed paired t-tests with Bonferroni correction were used. * = P<.0042; ** = P<.001. The symmetric error bars in both (A) and (B) represent two standard deviation units in length.

An increase in PFI was seen for all subjects following exposure to stimulation; however, the magnitude of the response was highly variable between individuals (Fig. 5). The magnitude of the peak response to stimulation ranged from an increase of 3.7% above baseline measured in the subject with the smallest response to stimulation, to a 14.4% increase in PFI over baseline in the subject with the greatest response to stimulation. The time at which the maximal response was observed also varied between subjects, occurring 30 seconds into stimulation for 3 subjects, and 45 seconds into stimulation for the remaining 2 subjects.

The increase in PFI following light stimulation was transient. In comparison to measurements obtained during baseline, PFI increased 6.1±4.7% (p = .001) during the first minute of pattern stimulation. In the second minute of pattern stimulation, PFI increased 2.0±5.4% (p = 0.275) above baseline. In the minute following cessation of pattern stimulation, PFI was 0.4±6.0% (p = 0.896) greater than baseline.

The increase in PFI was statistically significant at 15 seconds, 30 seconds and 45 seconds into stimulation (Table 1). The largest relative change to flow index was observed at 45 seconds into stimulation. However, the highly significant change to parafoveal retinal flow index was found after 30 seconds with stimulation. The PFI was not significantly different from baseline during the second minute of stimulation.

**Table 1:** Time Course of PFI Response.

	Stimulation on							
Time (sec)	15	30	45	60	75	90	105	120
Change from baseline	5.6%	7.1%	7.2%	4.4%	3.2%	1.7%	2.1%	0.9%
SD	4.4%	4.4%	5.1%	5.1%	5.0%	5.1%	5.2%	6.9%
P-value†	0.002	<0.001	0.001	0.018	0.070	0.336	0.262	0.782
	Stimulation off							
Time (sec)	15	30	45	60				
Change from baseline	0.7%	0.7%	0.0%	0.3%				
SD	6.0%	6.3%	6.6%	6.2%				
P-value	0.807	0.797	0.950	0.936				

SD, standard deviation; † Paired t-test

## Discussion

OCT has traditionally been used for structural imaging of the eye and has become important clinically in the evaluation of diseases such as glaucoma and age related macular degeneration (AMD) [30]–[32]. However, the applications for OCT are not limited to structure. We recently developed OCT angiography with SSADA as a noninvasive functional imaging tool to assess blood flow in the eye. Flow in vessels is measured with flow index, a composite index that reflects both flow velocity and vessel area [12]. In this study, we investigated the ability of our technique to evaluate neurovascular coupling in the retina using flow index as a measurement to quantify blood flow.

The evaluation of neurovascular coupling may be useful in the study of retinal diseases and generalized vascular dysfunction. Many ocular diseases such as glaucoma and diabetic retinopathy present signs of vascular abnormality [33], [34]. There have been previous studies that have shown an impaired vascular response to flicker light in both glaucoma and diabetic retinopathy patients [35]–[37]. As an easily accessible component of the cerebral microcirculatory, retinal vessels are also able to reveal vascular dysfunction in a variety of other health disorders. Several studies have shown a decreased retinal vascular response to light stimulation in hypertensive patients, obese subjects, and chronic smokers [38]–[40]. Thus, the ability to evaluate functional abnormalities, along with microstructural measurements, would increase the clinical utility of OCT.

Previous studies have shown increased retinal vascular caliber and blood flow following exposure to light stimulation. The magnitude of the maximal response increase measured with OCT angiography derived PFI (7.2%) was larger than previously measured vascular caliber changes, but smaller than known flow responses. Using fundus photography, Formaz *et al*. found a 4.2% increase in retinal artery diameter and a 2.7% increase in retinal vein diameter after exposure to diffuse luminance flicker [41]. Michelson *et al*. found a 46% increase in retinal capillary blood flow using SLDF, while Garhofer *et al.* found a 59% increase inretinal artery flow and a 53% increase in retinal vein flow when combining LDV with a retinal vessel analyzer [6], [7]. When the response to flicker was measured with Doppler OCT, Wang *et al*. found a 22.2% increase in total retinal blood flow [8].

In our study we chose to use an 8 Hz reversing checkerboard pattern rather than a flickering light to stimulate the retina. The reduced luminance and constant illumination of the pattern stimulation provided greater comfort to the subject during light stimulation, which allowed for a longer period of stimulation. At 2 minutes, the duration of light stimulation used in our experiment was twice the length of the longest stimulation period we encountered in previous studies that used flickering light [7]. Our expanded time course revealed the transient nature of the flow response to visual stimulation. The longest light stimulation duration we found in the literature was a 60 second flicker stimulation performed by Garhofer *et al*, during which increased retinal blood flow following flicker activation was sustained until the stimulation was turned off [7]. Sustained blood flow increase was also observed in shorter time courses that used 40 second and 20 second flicker periods [39], [42]. In our study, the largest and most significant PFI change from baseline measurements was observed 30–45 seconds into stimulation. No significant PFI changes were observed during the second minute of stimulation. Since the subjects received dilating eye drops, the adaptation was not due to the pupillary response. Light adaptation by photoreceptors is a likely explanation of the time course of the vascular response. Previous studies [43], [44] have shown that foveal light sensitivity decreases after the onset of light stimulation, reaches a minimum within 60–100 seconds, and finally reaches steady state after several minutes. This adaption response had been tested in the illumination range of ∼0.0002 - ∼0.2 lumen/cm^2^, which encompasses the stimulus strength we used. This cone light adaptation time course is quite similar to the parafoveal blood flow response we found in this study. Other adaption mechanisms in retinal neurons and blood vessels may also affect the vascular response that we observed.

A limitation of OCT angiography is that flow in inner retinal vessels project variable shadows on deeper reflective layers, causing decorrelation of deeper voxels that cannot be distinguished from true blood flow. This projection artifact prevents true volumetric flow measurement. Therefore it is necessary to compute the flow index on 2-dimensional angiograms formed by maximum flow projection. The maximum flow projection procedure may ignore some small deep retinal vessels lying under larger inner retinal vessels. Since retinal vessels are relatively sparse (only a small fraction of total retinal area is occupied by vessels), this discounting of overlapping vessels should have a relatively small effect on the computation of the flow index.

Another limitation associated with the technique is the possibility erroneous decorrelation introduced though eye movement artifacts while capturing scans. We attempted to minimize this effect by instructing subjects to fixate upon a projected fixation target that persisted through the entirety of each scan session. Additionally, our analysis of flow index in the FAZ showed that FFI was close to zero during baseline, 30 seconds into stimulation, and 30 seconds post-stimulation. The FAZ is a region void of blood vessels, and therefore any signal shown in the area can be attributed to eye motion artifacts and background noise. Since there were no significant differences between FFI measured during baseline, when stimulation was turned on, and when stimulation turned off, it suggests that PFI dynamics measured in our experiment were unlikely to have been influenced by eye motion artifacts.

The strength of OCT angiography lies within the high repeatability of baseline flow index measurements (CV 1.3% within a sequence and 2.1% between sequences). This allowed for the detection of small changes from baseline, and compares favorably to LDV (16–34% CV), SLDF with HRF (4.92–7.74% CV), and Doppler OCT (10.9% CV) [45]–[47]. However, the high variability of the response magnitude among normal subjects may limit the utility of this technique in differentiating abnormally low responses in individual patients.

## Conclusion

In summary, we have demonstrated for the first time the feasibility of using OCT angiography to measure the parafoveal retinal vascular response to visual stimulation. This technique may be useful for the study of ocular physiology and pathophysiology.
